# Effective treatment of cancer metastasis using a dual-ligand nanoparticle

**DOI:** 10.1371/journal.pone.0220474

**Published:** 2019-07-29

**Authors:** Gil Covarrubias, Felicia He, Shruti Raghunathan, Oguz Turan, Pubudu M. Peiris, William P. Schiemann, Efstathios Karathanasis

**Affiliations:** 1 Department of Biomedical Engineering, Case Western Reserve University, Cleveland, Ohio, United States of America; 2 Case Comprehensive Cancer Center, Case Western Reserve University, Cleveland, Ohio, United States of America; Brandeis University, UNITED STATES

## Abstract

Metastasis is responsible for the majority of deaths of breast cancer patients. While cytotoxic drugs are available with high potency to kill breast cancer cells, they are not designed to specifically seek and navigate in the dynamic and continuously changing microenvironment of metastatic disease. To effectively delivery chemotherapeutic agents to metastasis, we designed a dual-ligand nanoparticle loaded with doxorubicin by using two different types of ligands targeting EGFR and α_v_β_3_ integrin. Metastatic cancer cells continuously change resulting in heterogeneity even across adjacent micrometastatic regions with variable expression of these targetable receptors. Using a mouse model of breast cancer metastasis, in vivo and ex vivo imaging showed that both EGFR and α_v_β_3_ integrin-targeting were required to reliably direct the nanoparticle to metastasis and capture the spread and exact topology of the disease. Survival studies compared the anticancer efficacy of the standard drug, EGFR-targeting nanoparticle, α_v_β_3_ integrin-targeting nanoparticle and the dual-ligand nanoparticle. While all the other treatments produced moderate therapeutic outcomes, treatment with the dual-ligand nanoparticle yielded significant improvement and event-free survival in a mouse model of breast cancer metastasis.

## Introduction

Triple-negative breast cancer (TNBC) exhibits a very high risk of recurrence, resulting in disproportional mortality among breast cancer patients [1]. A total of 12–17% of newly diagnosed early breast cancers are TNBCs, corresponding to over 172,000 patients diagnosed annually worldwide [2, 3]. Compared to other subtypes of breast cancer, metastatic relapse will occur in the majority of these patients following treatment [4, 5]. Further, distant metastatic recurrence of TNBC (*m*TNBC) tends to occur in visceral organs, including the lungs, liver, and brain [4, 5]. Due to its metastatic phenotype and characteristically high recurrence rate, nearly all women with metastatic TNBC will eventually die of their disease. This stems from the fact that traditional systemic therapies are not designed to specifically seek out and navigate into the hard-to-reach microenvironment of metastatic disease.

Since there are no targeted therapeutics available for TNBC, the standard-of-care is primarily based on systemic chemotherapy. While cytotoxic drugs are designed and selected based on their potency to kill cancer cells, they do not take under consideration the dynamic and continuously changing microenvironment of metastatic disease [6, 7]. Even worse, the early stages of metastatic disease involves tiny colonies of cancer cells buried in healthy tissues [8], which makes early micrometastasis very challenging to target and differentiate from healthy tissues using drugs in their standard form. To increase drug delivery to tumor sites, targeting ligands have been used to direct deposition of drug-loaded nanoparticles to tumors that overexpress cancer-specific targetable receptors including folate, EGF, HER2, and integrin receptors. However, cancer cells at early micrometastatic sites evolve and continuously change the expression of these targetable receptors [9–18].

**Fig 1:**
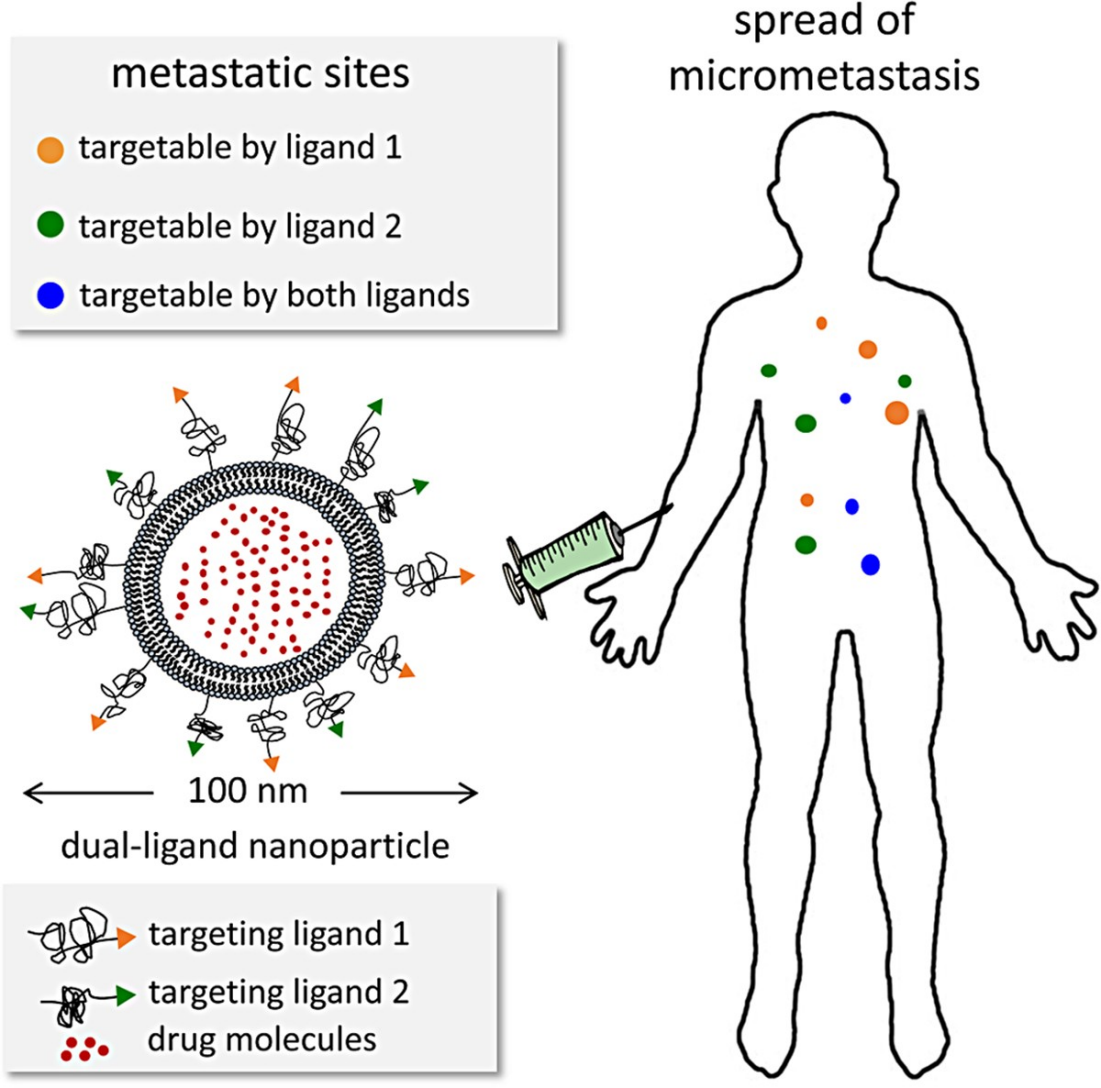
Illustration of the multi-targeting concept shows a dual-ligand nanoparticle targeting the dynamic nature of metastatic disease.

Recently, we developed targeting schemes using multi-ligand nanoparticles to account for the spatiotemporal changes in the expression patterns of targetable receptors in metastasis [15, 19, 20]. Due to their nanoscale size, we showed that nanoparticles are ideal for incorporating more than one types of ligands on their surface at sufficiently high ligand density to achieve accurate targeting of their corresponding targeted receptor. By decorating the surface of the nanoparticle with more than one types of targeting ligands, multi-ligand nanoparticles exhibited highly precise targeting of different subsets of metastasis that predominantly express different targetable receptors at any given time that were otherwise missed by single-ligand strategies. For instance, a dual-ligand nanoparticle targeting EGFR and α_v_β_3_ integrin can achieve a nearly 2-fold higher deposition into breast cancer metastasis in the lungs than its single-ligand nanoparticle counterparts [19]. We should mention that non-targeted nanoparticles achieve significantly lower deposition in metastasis than their single or dual-ligand targeting nanoparticle counterparts. This stems from the fact that the endothelium of early metastasis is not as leaky as observed in primary tumors. Our earlier studies focused on the imaging and diagnostic application of multi-ligand nanoparticles. Here, we exploit a dual-ligand drug-loaded nanoparticle for treatment of breast cancer metastasis (**Fig 1**). We selected a dual-ligand system using two peptides that target 1) EGFR, which is an overexpressed receptor on TNBC cells [21–29], and 2) α_v_β_3_ integrin, which a receptor involved with the leukocyte adhesion cascade that circulating TNBC cells use to attach to the endothelium of future metastasis [30–34]. Considering the topology of these two receptors, the target sites involve surface receptors on metastatic foci resident on the remodeled endothelium of micrometastasis [35–37]. As our nanoparticle, we chose the liposome, which is an all-purpose, highly versatile drug carrier with a long clinical history. Using a mouse model of metastatic TNBC, we demonstrate that the dual-ligand targeting scheme leads to precise and effective delivery of a clinically used cytotoxic nanoparticle (*i*.*e*., liposomal doxorubicin) to different metastatic sites, which are typically missed by the free unmodified drug or single-ligand drug-loaded nanoparticle.

## Materials and methods

### Nanoparticle fabrication

To prepare DSPE-PEG-ligand conjugates, the c(RGDfC) [35] or the CYHWYGYTPQNVI [38] peptide (Peptides International) was conjugated to DSPE-PEG-NH_2_. Using the cross-linker sulfo-SMCC (Thermo Fisher Scientific), the amine of DSPE-PEG-NH_2_ reacted for 2 h with the thiol of the cysteine residue on the peptides. Sulfo-SMCC contains an amine-reactive *N*-hydroxysuccinimide (NHS ester) and a sulfhydryl-reactive maleimide group to form stable amide and thioether bonds. To guarantee complete conjugation of DSPE-PEG-NH_2_, the peptide was at a 2-fold molar excess over the PEG. To remove unreacted peptide, the conjugates were dialyzed for 1 day against PBS. The completion of the reaction was confirmed using thin layer chromatography (TLC). TLC was carried out on silica gel coated fiber sheets using a mixture of CHCl_3_/MeOH as the mobile phase. More details can be found in previous publications [15, 19].

We prepared 100-nm liposomes encapsulating doxorubicin (DOX) using standard intraliposomal stabilization technology [39–41]. A lipid composition of DPPC, cholesterol and DSPE-PEG(2000)-ligand in the molar ratio of 60-X:40:X was used. For the single-ligand or dual-ligand nanoparticle variants, X was 2.5 or 5%, respectively. An equal ratio of the two DSPE-PEG-peptide conjugates was used in the case of the dual-ligand nanoparticle. The lipids were dissolved in ethanol and hydrated with 300 mM ammonium sulfate at 60°C followed by sequential extrusion in a Lipex Biomembranes Extruder (Northern Lipids, Vancouver, Canada). Finally, DOX was loaded into the liposomes across a gradient of the inner phase (pH 4.0) and the extraliposomal phase (pH 7.5). Following dialysis to establish an ammonium sulfate gradient, the liposome suspension was mixed with DOX for 30 min at 60°C. The liposomes were dialyzed against PBS for 1 day using a 100 kDa MWCO dialysis tubing (Spectrum Laboratories, CA). The size of the nanoparticles was characterized using dynamic light scattering (DLS, Brookhaven Instruments). The number of peptides on each nanoparticle variant was measured using a direct protein assay (Bio-Rad Protein Assay using Coomassie Blue G-250 dyes).

The nanoparticle variants were labeled with either an NIR fluorophore (Vivotag-S 680 or 750) or an Alexa fluorophore (Alexa 647 and 750), which contained an NHS functional group (Perkin Elmer) [15]. The fluorophore was conjugated directly onto the lipid 1,2-Distearoyl-sn-glycero-3-phosphoethanolamine (DSPE) in chloroform at 55°C in the presence of triethylamine. A 2-fold molar excess of the fluorophore was used over DSPE. Thin layer chromatography confirmed completion of the reaction. Following evaporation of the solvent, the lipids were used as part of the lipid matrix at 2.5 mol%. The unreacted fluorophore was eventually removed after formation of the liposomes using dialysis. The final level of the fluorescent label of liposome variant was measured using the Fluorescence Molecular Tomography (FMT) or the Spectrum In Vivo Imaging System (IVIS, Perkin Elmer).

### Animal model

All animal studies were conducted under a protocol approved by the Institutional Animal Care and Use Committee (IACUC) at Case Western Reserve University. The well-being of the animals took priority over precise measurements in decisions regarding euthanasia or other interventions. We used a mouse-syngeneic tumor model based on the D2.A1 breast cancer cells. The D2.A1 cancer cells were obtained from Dr. Fred Miller (Barbara Ann Karmanos Cancer Institute, Detroit, MI; ref. [42]). The D2.A1 cells stably expressed firefly luciferase and green fluorescent protein (GFP). Female BALB/c mice were injected *via* the tail vein with 5 × 10^5^ D2.A1 cells. After tumor implantation, mice were randomized into groups for subsequent studies. Bioluminescence imaging was performed every 3–7 days to monitor the progression of metastatic disease. Images were collected 10 min after intraperitoneal administration of 200 μl of D-luciferin (10 mg/ml) using an IVIS Spectrum system. Bioluminescence imaging (BLI) was performed every 3–7 days until the terminal point of the study. The animals were closely monitored on a daily basis to ensure they did not suffer adverse effects resulting from tumor inoculations.

### Animal research ethics statement

All animal procedures were conducted under a protocol approved (#2015–0116) by the Institutional Animal Care and Use Committee (IACUC) at Case Western Reserve University. The well-being of the animals took priority over continuation of planned interventions. All animals received standard care, including ad libitum access to food and water, a 12/12 light/dark cycle, appropriate temperature and humidity. All animals received standard care ensuring proper protocol guidelines were followed. The animals were closely monitored on a daily basis to ensure they did not suffer adverse effects resulting from tumor inoculations. The well-being of the animals took priority over precise measurements in decisions regarding euthanasia. All procedures were conducted using anesthetic to minimize pain and distress. The inhalant anesthetic, isoflurane, was used as the primary anesthesia in our experiments. However, developing tumors may ultimately result in some level of distress or discomfort in these mice. If, during the time following tumor inoculation the animal showed signs of post-procedure pain, the animal was euthanized. The research team, as well as the veterinary team of the animal facility, diligently monitored the condition of the animals, and removed any animal exhibiting signs of pain or distress as soon as humanly possible. When an animal showed distress or stopped eating and drinking (visually evaluated or there was a 15% loss of body weight), the animal was immediately euthanized. If it was observed that the tumor became 10% of the body mass of the animal or if there were changes in grooming, weight, behaviors, or kyphosis, the animal was immediately euthanized. Additionally, if we observed that an animal was suffering from inactivity, prostration, labored breathing, sunken eyes, hunched posture, piloerection/matted fur, unresolving skin ulcers, abnormal vocalization when handled, emaciation or anorexia, the animal was immediately euthanized. In all cases euthanasia was carried out in a CO_2_ chamber. Euthanasia was confirmed by cervical dislocation.

### Fluorescence *in vivo* and *ex vivo* imaging

Following injection of a cocktail of the two single-ligand nanoparticle variants, FMT imaging was performed at multiple time points after (t = 0, 30 min and 3, 24 h). The cocktail contained equal number of particles of RGD-targeted and EFGR-targeted nanoparticles (RGD-NP and EFGR-NP). The mice were injected with a dose containing ~5.3 × 10^11^ nanoparticles of each formulation. For the *in vivo* imaging studies, the nanoparticle formulations were labeled with a different NIR fluorophore. Using phantoms of each formulation, the FMT was calibrated to take quantitative deposition measurements. For the *ex vivo* imaging studies, the IVIS Spectrum system was used to image the lungs *ex vivo*. After injection of a cocktail containing the two single-ligand nanoparticle variants, the animals were anesthetized with an IP injection of ketamine/xylazine and transcardially perfused with heparinized PBS followed by 4% paraformaldehyde in PBS. After the organs were explanted, the lungs were precisely sliced in 500 μm sections using a mouse organ slicer. We have confirmed that negligible attenuation of fluorescence signal occurs through the 500-μm tissue thickness at the selected excitation wavelengths [19]. All the lung slices of each animal were imaged with the IVIS system to quantitatively assess the deposition of the various targeted nanoparticle formulations in lung metastasis. Using calibrations from phantoms of the fluorescently labeled nanoparticles, the signal from each lung slice was quantified, then added together for the entire lung and finally converted to total accumulation of nanoparticles. As control, we used lungs from saline-injected animals to subtract background fluorescence at the selected excitation wavelengths.

### Histological evaluation

Histological analysis was performed to evaluate the microdistribution of fluorescently labeled RGD-NP and EGFR-NP in metastasis in the lungs of mice. Mice were anesthetized with an IP injection of ketamine/xylazine and transcardially perfused with heparinized PBS followed by 4% paraformaldehyde in PBS. Organs were explanted and post-fixed overnight in 4% paraformaldehyde in PBS. The tissues were soaked in 30% sucrose (w/v) in PBS at 4°C for cryosectioning. Serial tissue sections of 12 μm in thickness were obtained. Using a fluorescence microscope, the tissue sections were imaged directly for green (GFP-expressing cancer cells), and the Alexa 647 and 750 fluorophore (nanoparticles). The tissue sections were imaged at 5, 10 or 20x on a Zeiss Axio Observer Z1 motorized FL inverted microscope.

Immunohistochemistry was performed to evaluate the expression of α_v_β_3_ integrin and EGFR in D2.A1 metastasis in the lungs. Serial tissue sections were stained with the nuclear stain DAPI and the specific antibody for α_v_β_3_ integrin or EGFR.

### Survival study

Mice bearing D2.A1 metastasis were treated with EGFR-NP, RGD-NP or dual-ligand NP *via* tail vein injection at a dose of 7.5 mg/kg DOX at days 3, 4 and 5 after tumor inoculation. Control animals were treated with free DOX at a dose of 2 or 7.5 mg/kg. The tumor growth was allowed to progress until the animals showed changes in grooming, weight, behaviors, at which point animals were euthanized in a CO_2_ chamber. Time of death was determined to be the following day.

### Statistical analysis

Statistics were performed in Prism version 7 for Mac (GraphPad Software, La Jolla, CA, USA). All the experiments were performed in triplicates unless stated otherwise. Data are represented as mean±s.d. Statistical significance between survival curves was determined using the log-rank (Mantel-Cox) test. In cases where data met the assumptions necessary for parametric statistics, analysis of differences between two groups was performed using two-tailed Student’s t-test assuming equal variance. Data from three or more groups were analyzed with a two-way analysis of variance (ANOVA) that was corrected for multiple comparisons using the Holm−Sidak method.

## Results

### Synthesis of nanoparticle variants

We synthesized a 100-nm liposomal nanoparticle in a manner similar to our previously published work [15, 19]. Using remote loading, the nanoparticle’s cargo was measured to be 0.2 mg DOX per 1 mg of lipids. The stability of the drug encapsulation was confirmed by dialyzing the formulation against PBS at 37°C. The drug leakage was less than 5% of the total encapsulated drug after 24 h. Using dynamic light scattering, the size of the nanoparticles was uniform with an average diameter of ~105 nm (with a polydispersity index of 0.03).

To be detectable in the *in vivo* imaging studies, the nanoparticle was labeled with an NIR fluorophore using Vivotag-S 645 or 680 or 750 and the Alexa 647 or 750, which contained an NHS functional group [15]. The fluorophore was conjugated directly onto the lipid 1,2-Distearoyl-sn-glycero-3-phosphoethanolamine (DSPE). To ensure complete conjugation of DSPE with Vivotag, a 2-fold molar excess of fluorophore was used over DSPE. Once thin layer chromatography (TLC) confirmed that the reaction was complete, the unreacted fluorophore was removed by dialysis. DSPE-fluorophore was used as part of the lipid matrix at 2.5 mol%. The final levels of the fluorescent label on each liposome were directly measured using Fluorescence Molecular Tomography (FMT) or the Spectrum In Vivo Imaging System. Stable fluorescence labeling was confirmed by dialyzing the nanoparticle formulations for 24 h dialysis resulting in no change in fluorescence signal.

To fabricate the targeting variants of the nanoparticles, the α_v_β_3_ integrin-targeting peptide *c(RGDfC)* and the EGFR-targeting peptide *CYHWYGYTPQNVI* were linked on the distal end of the PEG(2000)-NH_2_ of the parent nanoparticles. The two DSPE-PEG-peptide conjugates were prepared according previously established methods [19]. We prepared three nanoparticle variants including the EGFR-targeting nanoparticle (EGFR-NP), the α_v_β_3_ integrin-targeting nanoparticle (RGD-NP) and the dual-ligand nanoparticles (dual-ligand NP). The number of peptides on each nanoparticle variants was determined using direct protein assays (Bio-Rad Protein Assay using Coomassie Blue G-250 dyes), which showed that single-ligand nanoparticle contained ~2,000 peptides per particle whereas the dual-ligand variant had ~4,200 peptides per particle [19]. The zeta potential of the nanoparticles was measured to be slightly positive (~4 mV) [19].

### Targeting studies in the D2.A1 model

To evaluate the targeting and therapeutic performance of the dual-ligand nanoparticle, we used a mouse model of metastatic TNBC. Specifically, we used the D2-Hyperplastic Alveolar Nodules (HAN) series, which consists of various clonally related cell lines derived from the same premalignant murine hyperplastic alveolar nodule [43]. We used the metastatic D2.A1 cells that extravasate and initiate metastatic outgrowth in the lungs of syngeneic immunocompetent Balb/c mice. This is a well characterized model that provides a reliable assay system to thoroughly evaluate nanoparticles in metastatic TNBC in animals with intact immune systems [44, 45]. The D2.A1 cells stably expressed both fluorescent and bioluminescent reporter genes that allowed monitoring of the dissemination of metastatic disease in mice using *in vivo* imaging, *ex vivo* fluorescence imaging and histology. Mice were used in the targeting studies on day 15 after tail vein injection of the D2.A1 cells. At that point, bioluminescence imaging (BLI) indicated that metastasis was present in the lungs with a signal of ~3x10^7^ photons/sec. Immunohistochemical analysis showed that both EGFR and α_v_β_3_ integrin are overexpressed in lung areas with metastatic D2.A1 cells (S1 Fig).

**Fig 2:**
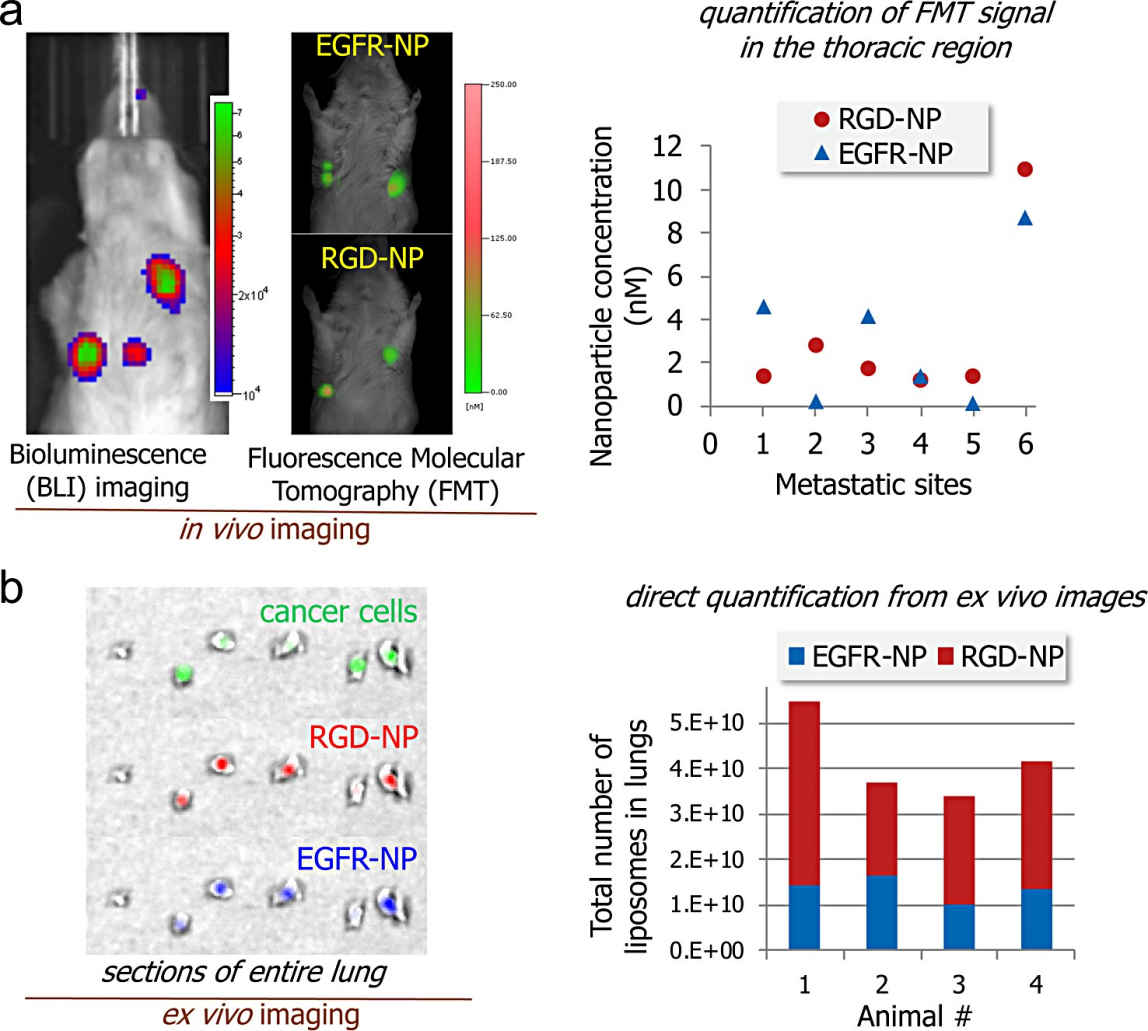
Targeting EGFR and α_v_β_3_ integrin in the D2.A1 mouse model of metastasis. **(a)** Bioluminescence imaging (BLI) shows the development of metastasis in the lungs (left). FMT in vivo imaging was performed 3 h after injection of a cocktail of EGFR-NP and RGD-NP. Using the different NIR fluorophores (Vivotag 680 and 750) on each nanoparticle variant, the fluorescence signal in each metastatic site of the FMT images was quantified for each formulation (n = 5 mice). On the basis of phantom measurements of each formulation using the FMT system, the fluorescence signal was converted to nanoparticle concentration. **(b)** In a different animal study, a cocktail of EGFR-NP and RGD-NP labeled with a different fluorophore (Alexa 647 and 750) was intravenously injected into animals with D2.A1 metastasis. After 3h from injection, lungs were perfused, excised, sectioned into thin slices of equal thickness and imaged ex vivo using an IVIS Spectrum system. The signal from each lung slice was quantified and summarized for the entire lung indicating the total number of each nanoparticle variant in the lungs of different mice with metastasis (n = 4 mice).

First, we compared the two single-ligand targeting variants, EGFR- and RGD-targeted nanoparticles in their ability to direct the nanoparticles to early metastasis. A cocktail of the two single-ligand nanoparticles was systemically injected into the same animals (n = 5 mice). The cocktail contained an equal number of nanoparticles of EGFR-NP and RGD-NP labeled with the NIR fluorophores Vivotag 680 and 750, respectively. Fluorescence molecular tomography (FMT) was used to perform *in vivo* imaging. FMT uses four NIR fluorescence channels that facilitate quantitative and simultaneous imaging of four different NIR fluorophores in the same animal [12, 46]. We have performed extensive studies that validate the quantitative accuracy of the results obtained from FMT imaging *in vivo* [15, 36–38, 46]. Each animal presented one or two metastatic sites in the lungs. **Fig 2**A shows an example of representative BLI and FMT images from the same mouse. **Fig 2**B summarizes the quantification of NIR fluorescence signal for the two single-ligand nanoparticle variants in each metastatic site. The data indicate that the two single-ligand formulations had different targeting performance varying from one metastatic site to the next. We should mention that the accumulation of non-targeted liposomal nanoparticles in metastasis was significantly lower than their EGFR- or RGD-targeting nanoparticle counterparts [19].

In another animal study, we sought to confirm the results from the *in vivo* imaging studies. To do so, we used the IVIS Spectrum system to image the lungs *ex vivo*. Our previous work has showed that EGFR and α_v_β_3_ integrin-targeting nanoparticles achieve maximum deposition in metastasis within 3 h after systemic administration [15, 19, 20, 35, 46]. A cocktail of EGFR-NP and RGD-NP labeled with different Alexa fluorophores (Alexa 647 and 750) was systemically injected (n = 4 mice). After 3h from nanoparticle injection, lungs were perfused, excised and precisely sliced in 500-μm sections using a mouse brain slicer. In previous studies, we confirmed that tissue sections with a thickness of 500 μm cause minor attenuation of signal from Alexa 647 and 750 fluorophores, which facilitated quantification of the concentration of the nanoparticle variants in lungs with metastasis. Representative images are shown in Fig 2B. It can be seen that the deposition of RGD-NP and EGFR-NP coincided with the locations of D2.A1 metastasis (green: GFP-expressing cells). Signal quantification shows a variable targeting performance from each nanoparticle variant. We then converted the fluorescence signal to percentile of the injected dose. Overall, 7.8% of the injected cocktail deposited at sites of metastasis with RGD-NP and EGFR-NP being 5.1 and 2.7% of the dose, respectively. In a previous study [19], the dual-ligand nanoparticle achieved an about two-fold higher deposition in metastasis than either single-ligand nanoparticle variant. To evaluate non-specific uptake of the nanoparticle by the lungs, the same cocktail of targeted nanoparticles was systemically injected in healthy animals resulting in negligible signals from the lungs.

### Evaluation of therapeutic efficacy

**Fig 3:**
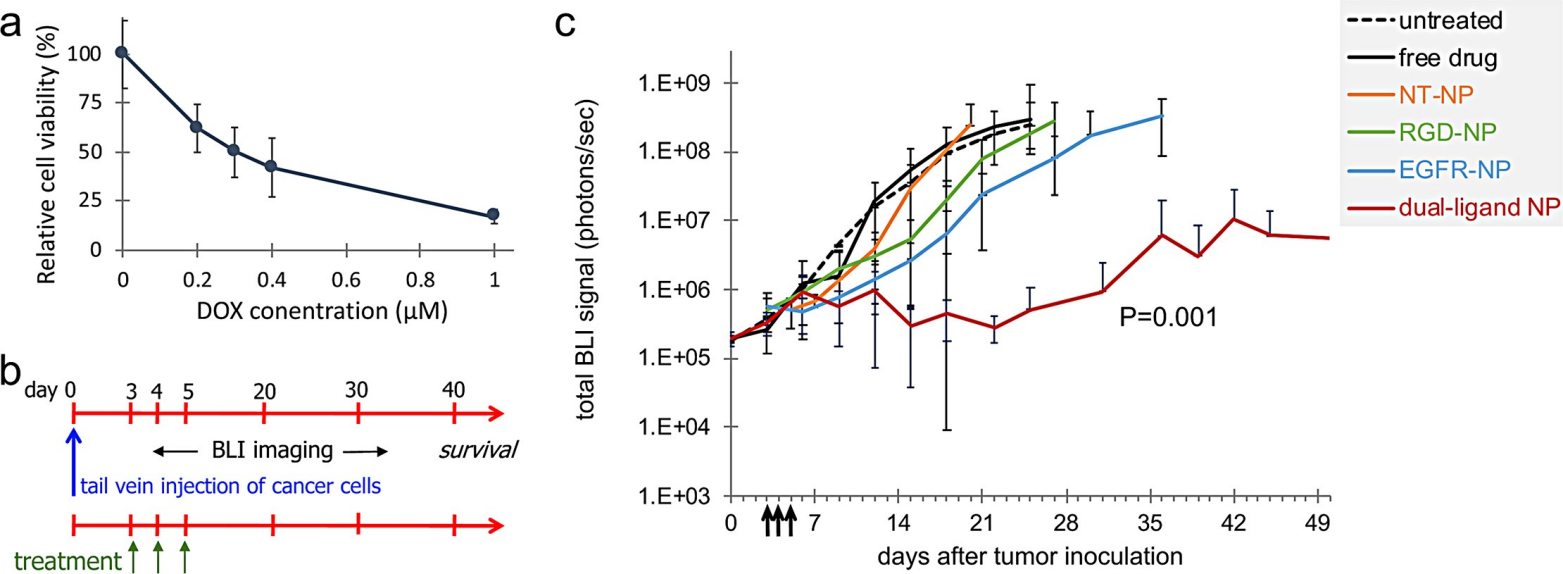
Treatment of mice with D2.A1 metastasis using dual-ligand nanoparticle loaded with doxorubicin. **(a)** The cytotoxicity of doxorubicin (DOX) was evaluated on D2.A1 cells. Cytotoxicity studies were performed by seeding D2.A1 cells at a density of 5 x 10^3^ cells per well. Cells were incubated with the treatment for 24 h at a concentration ranging between 0.2–1 μM DOX. After treatment application, the cells were washed three times with fresh medium and then incubated for 48 h at 37°C. The number of viable cells was determined using a formazan-based cell counting assay (CCK-8). Untreated cells served as live controls for normalization of the data. Data points represent group mean ± s.d. **(b)** The timeline and schedule of treatments are shown with respect to tumor inoculation. **(c)** The response of cancer metastasis to treatment was monitored using longitudinal BLI imaging. Quantification of the BLI signal in the thoracic region is shown for mice with D2.A1 metastasis treated at days 3, 4 and 5. In addition to untreated animals, treatments included non-targeted NP (NT-NP), RGD-NP, EGFR-NP, dual-ligand NP, and free DOX (n = 6–8 mice per treatment). The y-axis is in logarithmic scale. All nanoparticle formulations were administered at 7.5 mg/kg DOX (two-way ANOVA with repeated measures).

To assess the therapeutic efficacy of a drug-loaded nanoparticle with the capability of simultaneously targeting EGFR and α_v_β_3_ integrin, we fabricated a dual-ligand nanoparticle (dual-ligand NP), which contained an equal number of the EGFR and RGD-targeting peptides (total ~4,200 peptides per particle). Doxorubicin was the drug of choice due to the long clinical history of liposomal doxorubicin. First, we evaluated the cytotoxicity of DOX against the D2.A1 cells. As shown in **Fig 3**A, DOX demonstrated significant cytotoxicity against D2.A1 cells with the 50% inhibitory concentration (IC50) being 0.3 μM. This indicates that DOX has a strong anticancer activity against D2.A1 cells.

Fig 3B shows the timeline and schedule of treatments (n = 6–8 mice per treatment). As an initial metric of responsiveness of metastatic disease to the treatments, quantification of BLI signal in the thoracic region was used. Animals were treated at days 3, 4 and 5 after tumor inoculation with standard free DOX, the standard non-targeted nanoparticle (NT-NP), the two single-ligand nanoparticle variants or the dual-NP at a dose of 7.5 mg of DOX per kg bw. At the beginning of treatment (day 3), the BLI signal from the lungs of the animals was ~4x10^5^ photons/sec indicating the presence of metastasis. BLI imaging was performed every 3–4 days. As shown in Fig 3C, metastatic disease progressed rapidly in the case of the untreated group and animals had to be euthanized by day 25. Even though DOX is a highly potent cytotoxic agent, the group treated with standard DOX exhibited similar progression of the disease to the untreated group. Similarly, the NT-NP did not have any therapeutic effect on metastatic disease. On the contrary, metastatic outgrowth was delayed in the groups treated with either single-ligand nanoparticle variant with the EGFR-NP treatment being more effective. The BLI signal of the groups treated with RGD-NP and EGFR-NP reached the high value of 10^8^ photons/sec by day 27 and 30 respectively. Most notably, the entire group treated with dual-NP displayed very low BLI signal until day 30, at which point aggressive metastatic disease recurred only in a subset of the animals.

**Fig 4:**
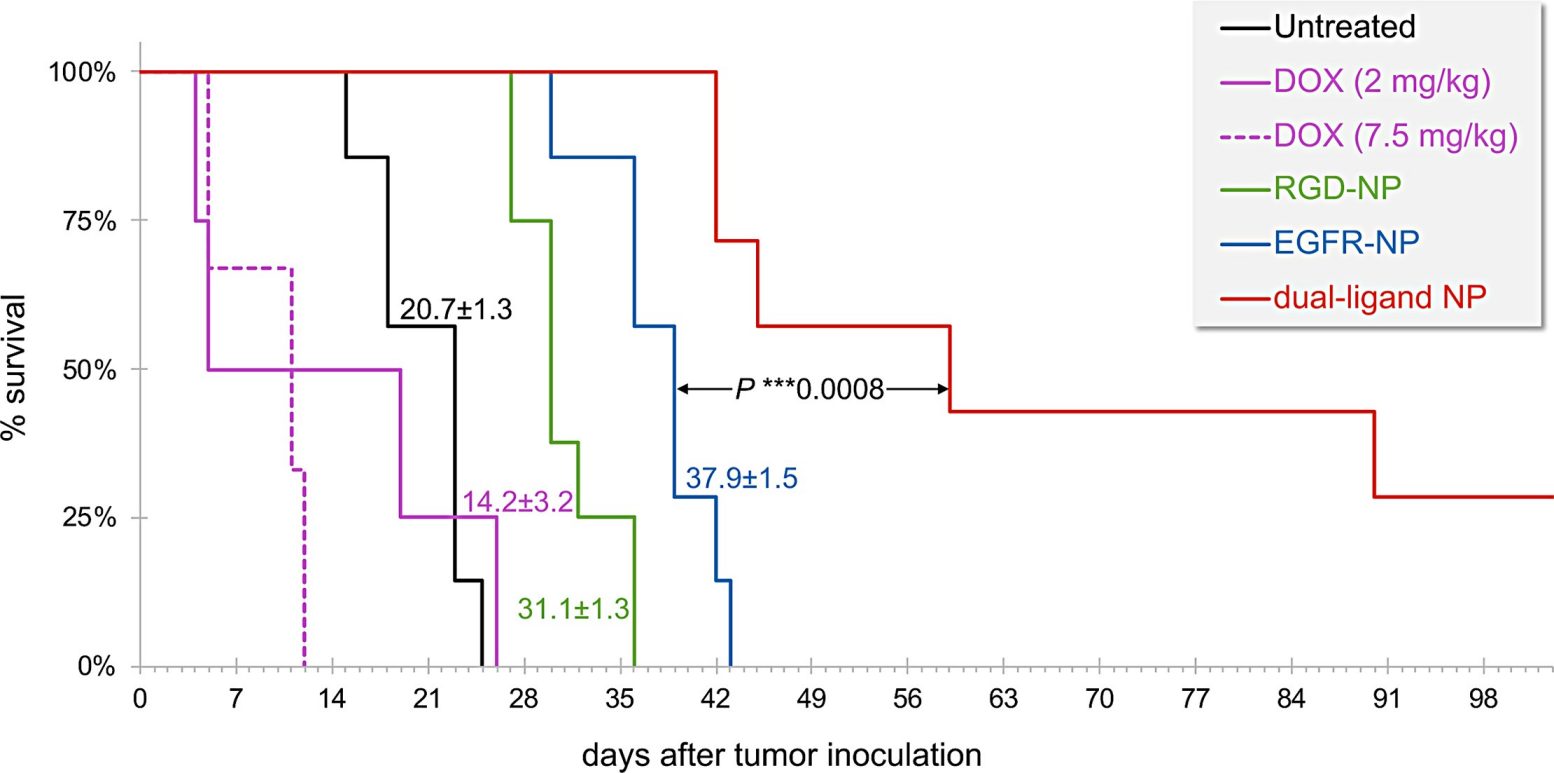
The survival time of metastasis-bearing mice treated with cytotoxic drugs was compared to untreated animals. The animals were treated at days 3, 4 and 5. The dual-ligand nanoparticle formulation was administered at 7.5 mg/kg DOX. In addition to the nanoparticle formulation, treatments included free DOX injected at 7.5 or 2.5 mg/kg (n = 6–8 mice per treatment). The difference between the survival curves of the dual-ligand NP and EGFR-NP-treated groups was assessed by the log-rank (Mantel-Cox) test.

In addition to BLI imaging, we compared the survival rates of the different treatment groups (**Fig 4**). The survival rate was in good agreement with BLI imaging. The treatments were generally well tolerated. The body weight change for representative groups is shown in S2 Fig. While exact longitudinal measurements of the group treated with the dual-ligand NP were not recorded, the weight of these mice was similar to the single-ligand NP treatments with a less than 10% loss immediately after treatment. All the groups treated with DOX-loaded nanoparticles maintained their body weight without any dramatic weight loss at the time of treatment. The survival of the untreated and free DOX-treated group (2 mg/kg) was comparable indicating the standard form of the chemotherapeutic drug had negligible therapeutic benefits. In the case of free DOX, we used a low and a high dose (2 and 7.5 mg/kg DOX). Notably, treatment with the high dose of DOX (7.5 mg/kg) affected negatively the survival of the animals, which can be attributed to the high toxicity of the treatment itself. The survival rates of the two single-ligand nanoparticle treatments were also in good agreement with the BLI data. Both targeted nanoparticles prolonged survival compared to the free drug treatment with the EGFR-NP being more effective. While 100% of the mice in the other groups did not survive more than 43 days, about a third of the dual-ligand NP-treated group was still alive at 120 days.

### Histological characterization

**Fig 5:**
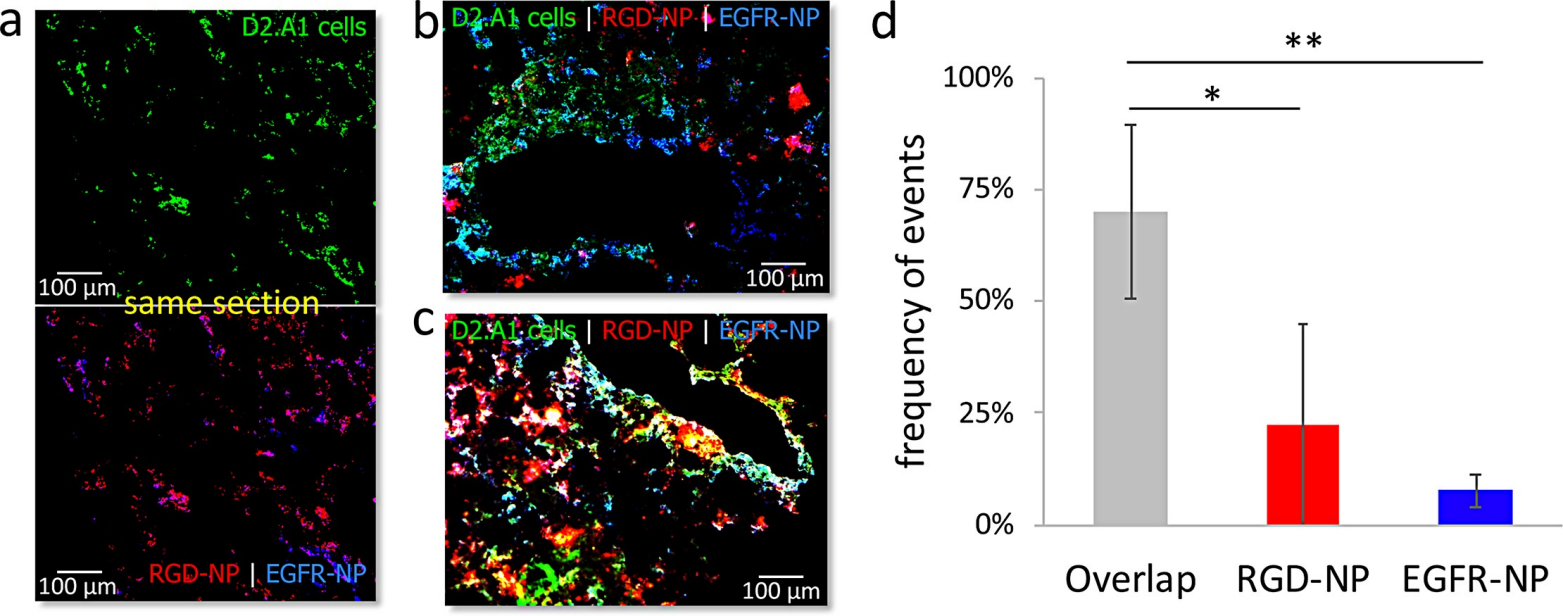
Histological evaluation of the microdistribution of RGD-NP and EGFR-NP nanoparticles in metastasis in the lungs of mice. **(a)** Representative fluorescence image of lung tissue shows dispersion D2.A1 metastatic cancer cells (top; 20X magnification). After 3 h from injection of a cocktail containing Alexa 350-labeled EGFR-NP and Alexa 568-labeled RGD-NP, the two targeting variants colocalized in locations with metastatic cancer cells (bottom). Different regions with metastatic cancer cells were predominantly targeted by **(b)** EGFR-NP or **(c)** RGD-NP (green: D2.A1 cancer cells; red: RGD-NP; blue: EGFR-NP). **(d)** A pixel-by-pixel quantification indicates individual events for EGFR and RGD-NP or their overlap (n = 3, grouped analysis ANOVA; correct for multiple comparisons using the Holm−Sidak method. *P* values: *0.024, **0.002).

We sought to histologically assess the deposition of RGD-NP and EGFR-NP in metastasis. We labeled EGFR-NP and RGD-NP nanoparticles with the Alexa 350 and 568 fluorophore respectively, which allowed both nanoparticles to be visualized in the same histological section using fluorescence microscopy. Mice bearing D2.A1 metastasis were injected with a cocktail containing an equal number of EGFR-NP and RGD-NP particles was injected to mice bearing D2.A1 metastasis. After 3 h from the injection of the cocktail, the lungs were perfused, excised, and processed for histology. **Fig 5** shows representative images. Both EGFR-NP and RGD-NP were predominantly deposited in locations with dispersion of metastatic cancer cells by targeting the near-perivascular regions and remodeled endothelium of metastasis (Fig 5A). While both EGFR-NP and RGD-NP colocalized in regions with metastatic cancer cells, some metastatic regions were primarily targeted only by EGFR-NP (Fig 5B) or RGD-NP (Fig 5C). Fig 5D shows a quantification of multiple histological sections indicating that frequency of individual events for EGFR-NP and RGD-NP or their overlap.

## Discussion

In this work, we explored the ability of multi-ligand targeting schemes to direct drug-loaded nanoparticles to breast cancer metastasis and its highly heterogeneous microenvironment. In previous studies [15, 19, 20], we employed and tested different combinations of peptides as ligands on nanoparticles that target EGFR, β_3_ and β_1_ integrins, P-selectin, and fibronectin. These different biomarkers represent different processes and stages of development of metastatic breast cancer [15, 47]. The processes include the molecular mechanisms that underlie the interplay between epithelial-mesenchymal transition (EMT) and its counterpart mesenchymal-epithelial transition and the processes that enable metastatic outgrowth and proliferative programs [47, 48]. By using various animal models of metastasis including 4T1, D2.A1, D2.OR and MDA-MB-231, those earlier studies showed that only multi-ligand nanoparticles were able to accurately target a broad spectrum of breast cancer metastasis either at dormancy or very early transient stages of aggressiveness [19]. Here, we selected two ligands that target α_v_β_3_ integrin and EGFR that exhibit spatiotemporal variability representing different cancerous activities and stages of metastatic development. The adhesion and attachment of circulating tumor cells to the endothelium of distant metastasis is mediated by α_v_β_3_ integrin present on both cancer and endothelial cells [31]. In addition to adhesion-specific markers, metastatic breast cancer cells carry a continuously varying overexpression of the cell-surface EGF receptor, which contributes to tumor invasiveness and metastasis [47, 49]. The targeting data for the single-ligand variants indicated that RGD-NP and EGFR-NP resulted in deposition at metastatic sites of 5.1 and 2.7% of the injected dose, respectively. While α_v_β_3_ integrin-targeting frequently led to higher nanoparticle deposition than EGFR-targeting, histological analysis illustrated the significant spatial variability between the two targeting variants, suggesting that a single-ligand formulation is not capable of capturing each and every region with metastatic cancer cells. This indicates that EGFR-NP and RGD-NP exhibited complimentary targeting of metastatic sites and their various tumor microenvironments.

Nanoparticles are highly suitable to multi-ligand schemes due to their ability to accommodate a high number of ligands on their surfaces and enhanced multi-targeting avidity as a result of geometrically enhanced multivalent attachment on the targeted receptor. Importantly, multi-ligand targeting schemes are not restricted to one nanoparticle type and can be adapted by most nanoparticle systems. To showcase the application of multi-ligand targeting, we selected a liposomal nanoparticle, because it is an all-purpose, versatile drug carrier for numerous types of drug molecules with high potential for clinical translation. More specifically, we rethought the 100-nm PEGylated liposome with a size, composition and drug cargo similar to that used in the clinic for patients with metastatic breast cancer. In typical scenarios, adjuvant chemotherapy is given to high-risk patients even though often the disease has not become clinically apparent. Here, we sought to address this unmet clinical need for metastasis-specific chemotherapy and improve treatment regimens by replacing a traditional nanoparticle antineoplastic agent with a safe, effective variant. The survival studies indicate that the dual-ligand liposome could identify clinically silent metastasis with a high degree of precision. It should be noted that the treatment started at an early time point of the metastatic progress, which represents the early stage of micrometastasis. Similar to the clinical experience, micrometastasis in its early and transient stages does not exhibit a fully developed mass and lacks angiogenic activity, which makes it inaccessible *via* passive accumulation through leaky vasculature. Due to efficient vascular targeting, we suggest that a long-term therapy can be administered to asymptotic high-risk patients to effectively establish remission and ultimately cure. Further, when we administered the cytotoxic drug in its free form at the same dose as the dual-ligand nanoparticle (*i*.*e*., 7.5 mg/kg), the treatment had adverse effects resulting in reduced survival, probably due to enhanced systemic toxicity.

This work shows that multi-ligand nanoparticles can successfully deliver drugs to the majority of metastatic sites and effectively treat this lethal disease. Overall, efficient and precise delivery of potent chemotherapy yielded significant improvement in event-free survival in mouse models of triple-negative breast cancer metastasis at a safe dose compared to typical clinical regimens.

## Supporting information

S1 Fig Histological evaluation. Immunohistochemistry was performed to evaluate the expression of α_v_β_3_ integrin and EGFR in D2.A1 metastasis in the lungs. Serial tissue sections were stained with the nuclear stain DAPI and the specific antibody for α_v_β_3_ integrin or EGFR.
(TIF)

S2 Fig Body weight progression. The average % change of body weight of mice bearing D2.A1 metastasis is shown after treatment with DOX-loaded nanoparticles (n = 6–8 mice per group), including the non-targeted NP (NT-NP), EGFR-targeted NP (EGFR-NP) and α_v_β_3_ integrin-targeted NP (RGD-NP).
(TIF)
