# Supplementary figures and images for: Effective treatment of cancer metastasis using a dual-ligand nanoparticle
Source: PLoS One. 2019 Jul 29;14(7):e0220474. doi: 10.1371/journal.pone.0220474 (PMC6663022; doi:10.1371/journal.pone.0220474)

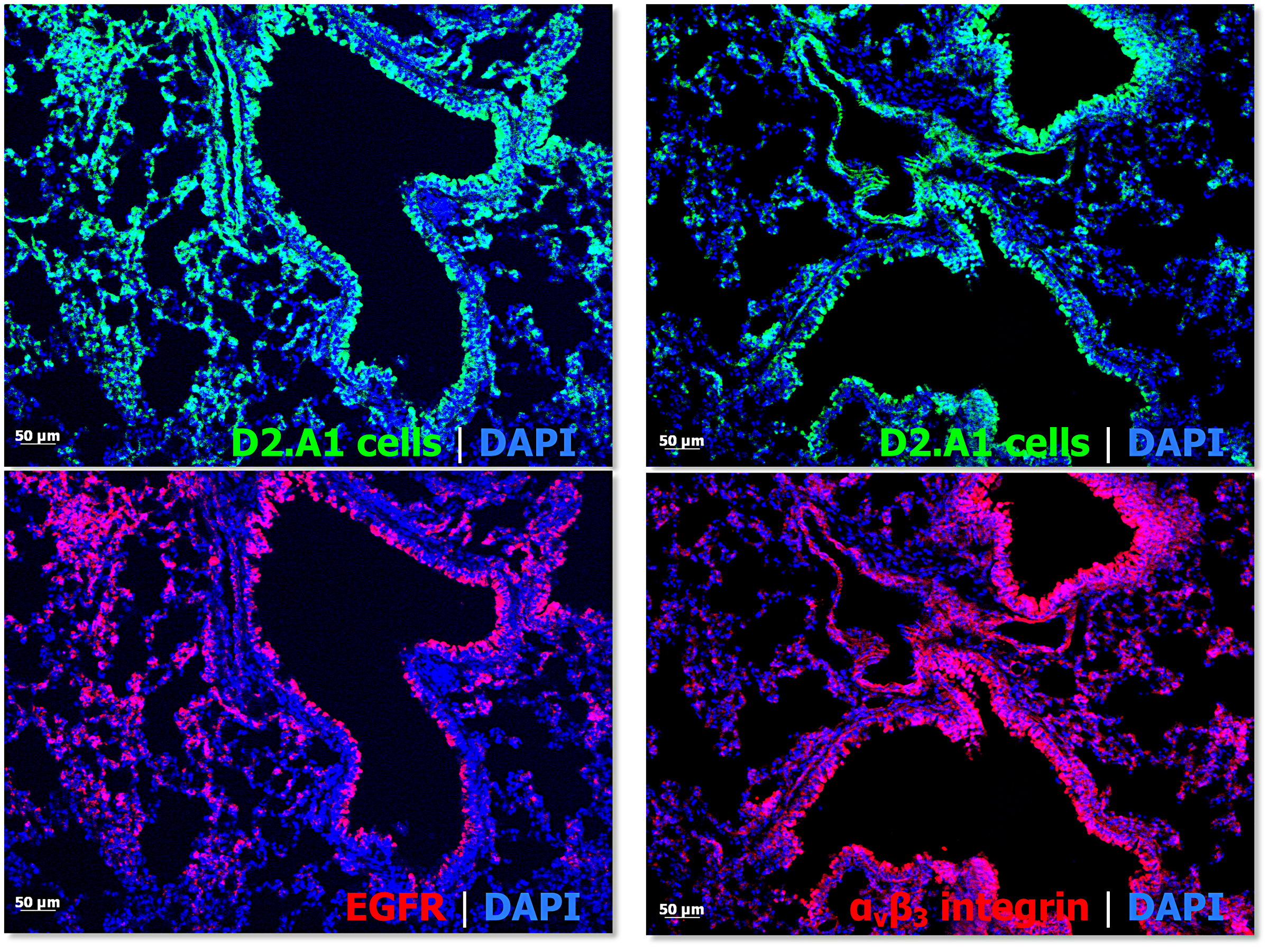

Supplement: S1 Fig — Immunohistochemistry was performed to evaluate the expression of αvβ3 integrin and EGFR in D2.A1 metastasis in the lungs. Serial tissue sections were stained with the nuclear stain DAPI and the specific antibody for αvβ3 integrin or EGFR. (TIF) [file pone.0220474.s001.tif]

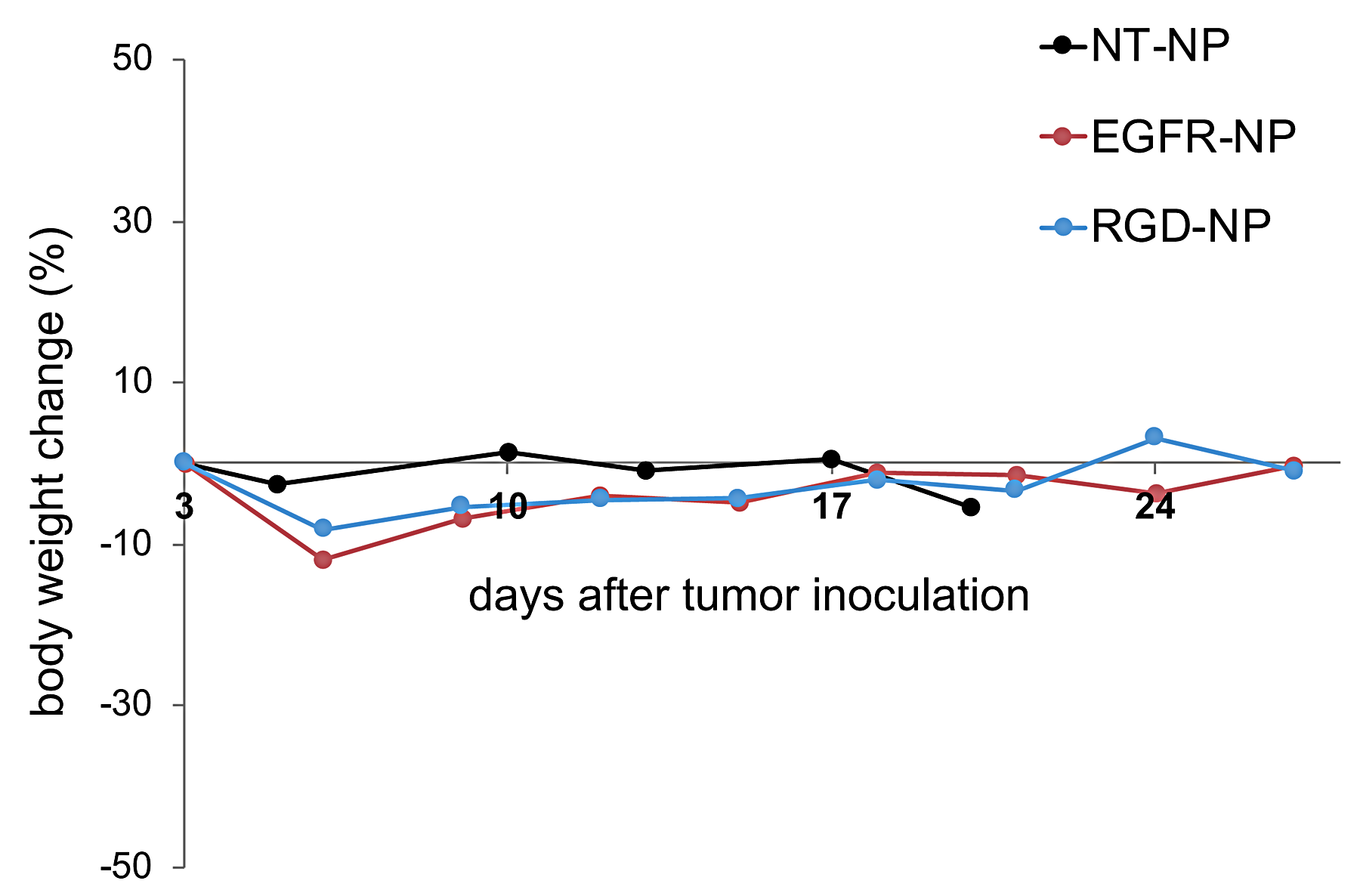

Supplement: S2 Fig — The average % change of body weight of mice bearing D2.A1 metastasis is shown after treatment with DOX-loaded nanoparticles (n = 6–8 mice per group), including the non-targeted NP (NT-NP), EGFR-targeted NP (EGFR-NP) and αvβ3 integrin-targeted NP (RGD-NP). (TIF) [file pone.0220474.s002.tif]
